# Protective Effect of *Catharanthus roseus* Extract on Cadmium-Induced Toxicity in Albino Rats: A Putative Mechanism of Detoxification

**DOI:** 10.3390/metabo12111059

**Published:** 2022-11-02

**Authors:** Mohammad Hashim, Hussain Arif, Baby Tabassum, Amin Arif, Ahmed A. Rehman, Shahnawaz Rehman, Rehnuma Khanam, Bushra Khan, Arif Hussain, Jameel Barnawi, Faris J. Tayeb, Naseh Algehainy, Faisal H. Altemani, Rashid Mir, Fahad M. Almutairi, Mohammad Fahad Ullah, Imadeldin Elfaki, Mohammad Rehan Ajmal

**Affiliations:** 1 Department of Biochemistry, S. S. Faculty of Science, Mohammad Ali Jauhar University, Rampur 244901, UP, India; 2 Toxicology Laboratory, Department of Zoology, Govt. Raza P. G. College, Rampur 244901, UP, India; 3 Department of Biochemistry, Faculty of life Sciences, Aligarh Muslim University, Aligarh 202002, UP, India; 4 School of Life Sciences, Manipal Academy of Higher Education-Dubai Campus, Dubai P.O. Box 345050, United Arab Emirates; 5 Department of Medical Lab Technology, Faculty of Applied Medical Sciences, Fahd Bin Sultan Research Chair, University of Tabuk, Tabuk 71491, Saudi Arabia; 6 Physical Biochemistry Research Laboratory, Biochemistry Department, Faculty of Science, University of Tabuk, Tabuk 71491, Saudi Arabia; 7 Department of Biochemistry, Faculty of Science, University of Tabuk, Tabuk 71491, Saudi Arabia

**Keywords:** cadmium, *Catharanthus roseus*, oxidative stress, comet assay, MDA, DPPH

## Abstract

Globally, people are highly affected by Cadmium (Cd), the most hazardous heavy metal. It has been implicated in various pathogeneses. Oxidative stress may be one the main reasons for Cd-induced disorders in the body. This article investigates the protective ability of *Catharanthus roseus* (CR) extract on oxidative stress in the kidney and liver of rats exposed to Cd. After 21 days, a significant increase in MDA concentration (6.81 ± 0.05), (6.64 ± 0.03) was observed in Cd-treated groups compared to the control (5.54 ± 0.02), (5.39 ± 0.04) for the kidney and liver, respectively, while significant changes were observed in the haematological parameters. Antioxidant enzymes, GPx, CAT, and SOD showed a significant decrease in their activity. We established that increasing the concentration of Cd in the presence of H_2_O_2_ was able to cause stand scission in pBR322 plasmid DNA, which may be due to the mediation of ROS generated in the process. The antioxidant ability of CR extract was tested in DPPH and H_2_O_2_ scavenging assay, depicted by the increase in the percentage inhibition. Upon treatment of CR extract to rats, MDA concentration was decreased for the kidney and liver compared to the Cd-treated groups. This was again confirmed by comet assay of both tissues, where the degree of cellular DNA breakage caused by Cd toxicity decreased significantly upon treatment with CR extract. Overall, the results suggest that Cd plays a major role as an effector metal ion, causing a decrease in the concentration and activity of AO enzymes and enhanced lipid peroxidation. ROS production resulted in oxidative DNA damage within the cell, whereas CR extract showed potential antioxidant activity against ROS-mediated DNA damage induced by Cd poisoning.

## 1. Introduction

In recent years, there has been increasing interest in the ecological effects and global population health concerns of environmental pollutants such as heavy metals. According to UNEP/GPA [1], the WHO and the European Parliament of Council, the occurrence of such metals in the ecosystem includes environmental sources such as agricultural, geogenic, and man-made sources such as electronic wastes, cosmetic products, pharmaceuticals and industrial and domestic effluents [2,3,4], which are considered hazardous to human life because they remain un-degraded [5,6]. According to Johnston [7], about 40,000–80,000 people around the world are still impacted by mercury poisoning; 200 million people are affected by lead poisoning; and approximately 250 million people are affected by cadmium (Cd) poisoning. Exposure to specific chemicals was responsible for the loss of 2 million lives and 53 million disability-adjusted life-years in 2019, according to the estimates that were published in the 2021 data supplement by the WHO [8]. Cd is an abundant, ubiquitous, nonessential, divalent, and most toxic industrial and environmental heavy metal that can accumulate in animals and human beings [9]. According to the United States Poison and Disease Registry, Cd is ranked sixth among toxic substances as being one of the most serious health risks to humans. Environmental Cd contamination has been a major cause of concern all over the world because of “itai-itai” disease, which was one of the first diseases caused by environmental pollutant-Cd poisoning in Japan during the 1950’s. Many international organisations have developed a set of criteria and guidelines to direct research into the harmful effects of Cd toxicity on human health [10]. In humans and animals, Cd absorption is firstly through inhalation, oral, and dermal exposure, in decreasing order of effectiveness [11]. Cd has been known to disrupt the endocrine and respiratory systems in humans and animals and may act as an oxidative stress inducer in cells, leading to nephrotoxicity and liver malfunction [12,13,14]. Reports suggest that Cd has a biological half-life of more than 15 years, and it can be retained in the liver and kidney for decades, causing deleterious effects on the cells [15,16]. According to Zhong [17] and Rafati [18], studies conducted on various cell lines showed a decrease in total glutathione levels and protein bound sulfhydryl groups by cadmium, which corresponds to the increased generation of reaction oxygen species (ROS) such as hydroxyl radicals, hydrogen peroxide, and superoxide ions [19,20]. The high levels of reactive oxygen species repeatedly result in increased lipid metabolism, more lipid peroxidation and modulation of intracellular oxidised states; DNA damage; altered gene expression and apoptosis. Our work demonstrates that Cd (a non-redox metal ion) induces DNA breakage in vitro and in vivo, similar to copper, a redox metal ion found in the nucleus that shows a Fenton type reaction and causes oxidative DNA breakage by generation of free radicals (ROS) under certain physiological conditions [21,22]. Although Cd is a non-redox metal, it can still cause DNA breakage, but the mechanism is not known. It would be significant to know whether Cd causes DNA damage either directly or indirectly. Therefore, we hypothesised that Cd could induce DNA breakage as a result of ROS production in the presence of H_2_O_2_ under certain conditions, which may lead to DNA damage. Previously, it has been reported that Cd^+2^ ions can react with nucleobases, nucleic metallothionein, and cause oxidative plasmid DNA breakage [23].

As reported, plant-based medicines and their constituents are being used rapidly in the treatment of various toxic diseases in human beings, whether directly or indirectly [24,25]. Medicinal plants, vegetables, and fruits, which are rich in phytochemicals such as terpenoid indole alkaloids, flavonoids, vitamins, and minerals, have many protective anti-oxidative effects against ROS and may act as alternative medicine to counter the harmful effects of heavy metal toxicity. However, the bioavailability of such plant-based constituents and their properties play an important role in their protection. In this study, we have tried to establish that exogenously supplemented CR extract can show protection against cadmium-induced toxicity. Common sadabahar, or *Madagascar periwinkle,* (CR), is an incessant plant belonging to the family Apocynaceae. Furthermore, it possesses a number of pharmaceutical roles of great medicinal importance, such as anti-carcinogenic, antioxidant, anti-inflammatory, anti-nephritic, and anti-bacterial properties [26,27,28]. Chemically, CR extract contains more than a hundred different terpenoid indole alkaloids (TIAs), flavonoids and saponins [29], among which vinblastine and vincristine are well-known, clinically used anti-carcinogenic alkaloids [30,31]. They are also known to protect cells from various intracellular cytotoxicity caused by heavy metal poisoning such as Cd [32]. The current in vitro/in vivo study aims to evaluate the antioxidant effect of CR extract via focusing on its ability to prevent oxidative DNA breakage caused on by Cd toxicity. These are conducted with the goal of improving our knowledge of the antioxidant effect of CR extract.

## 2. Materials and Methods

Eighty adult male albino rats (*Rattus norvegicus*) of the Wistar strain were selected from inbred colonies. All the rats were almost the same age and weight (150 ± 10 gm). They were acclimatised at room temperature with a 12 h dark/light cycle and fed a standard diet and water ad-libitum. After acclimatization, rats were randomly divided into four groups; one acute (1 day) and three sub-acute (7, 14 and 21 days). Each set has four groups containing one control (group A) and three tests (groups B, C, and D). Untreated animals in the control group received an equivalent volume of the vehicle by gavage. The doses of metals used in the present work were selected based on the previously done studies by Hashim [33]. The rats were maintained as per the latest guidelines of the committee for the purpose of control and supervision of experiments on animals (714/02aCPCSEA). Rats were anaesthetised after 24 h of the last dose, and organs were carefully removed from each animal and immediately used for the preparation of homogenates. The kidney was divided into cortex and medulla; both portions were homogenised separately in a glass Teflon homogenizer to prepare diluted homogenates in buffer (2 mM Tris-HCl, 50 mM mannitol, pH 7.5). Liver sections were similarly chopped, cleaned, and homogenised in a 10 mM Tris-HCl buffer. All the homogenates were divided into aliquots; these samples were either immediately used or stored at −80 °C for experiments.

### 2.1. Experimental Plant

CR was grown in a garden associated with our laboratory and ethanolic extract was prepared from its leaves. The leaves were dried in the shade, ground up roughly, and put in a soxhlet extractor at 55 °C with 70% ethanol as a solvent. The filtrate obtained by vacuum filtration was concentrated and dried using a vacuum evaporator under a controlled temperature (40–50 °C) [34]. Further safety trials were performed to determine the dose of extract and it was set to be 500 mg/kg body weight.

### 2.2. Haematological Parameters

Haematological parameters performed by manual methods included red blood cell count (RBC), white blood cell count (WBC), platelet count (PLT), haemoglobin concentration (Hb), packed cell volume (PCV), or haematocrit. PCV was measured by the micro-haematocrit method using capillary tubes, while RBC and WBC were measured manually using an improved Neubauer counting chamber. The platelet count was measured manually using a thin blood film stained with Leishman stain, and the haemoglobin concentration was determined by Shali’s method. Mean corpuscular volume (MCV) and mean corpuscular haemoglobin (MCH) were calculated using RBC, PCV, and [Hb] standard formulae from physiological and haematological textbooks [35].

The observations were analysed statistically using analysis of variance (ANOVA) followed by the Student’s Newmann–Keul’s (SNK) test. The obtained values were compared to the control and signified at the level of *p* < 0.01.

### 2.3. Hydrogen Peroxide Radical Scavenging Assay

The ability of the hydro-alcoholic extract of CR to reduce hydrogen peroxide was assessed by the method described by Gulcin [36]. In phosphate buffer, 40 mM H_2_O_2_ was dissolved (pH 7.4). Plant extract and ascorbic acid were dissolved in distilled water and added to a 40 mM hydrogen peroxide solution in increasing concentrations. Room temperature incubation lasted 30 min. Hydrogen peroxide absorbance was measured at 230 nm using a UV spectrophotometer against a blank phosphate buffer solution to prevent background. Ascorbic acid was a positive control for hydrogen peroxide in phosphate buffer. The experiment was performed in triplicate. The percentage scavenging of hydrogen peroxide of samples and ascorbic acid was calculated using the following formula.

$$\mathrm{Percentage}\;\mathrm{of}\;\mathrm{Hydrogen}\;\mathrm{peroxide}\;\mathrm{scavenging}\;\mathrm{activity}=\frac{Ao\;\;\;-\;\;Ai}{Ao}\times 100$$

where, Ao is the absorbance of the control and Ai is the absorbance of the plant extract and ascorbic acid.

### 2.4. DPPH (1,1-Diphenyl-2-picrylhydrazyl) Radical Scavenging Assay

In this assay, the assay is designed to determine the bleaching of a purple-coloured methanol solution by the DPPH radical. The radical scavenging activity was determined using method described by Abe with slight modification [37]. The hydrogen atom donating ability of the plant extractives was determined by the decolourization of a methanol solution of 2,2-diphenyl-1-picrylhydrazyl (DPPH). In the presence of antioxidants, DPPH produces a violet/purple colour in methanol solution and fades to shades of yellow. A total of 1 mL of 0.5 mM DPPH radical soluble methanol was added to the sample 500 μg/mL at increasing concentrations, followed by a phosphate buffer (pH 5.5). The mixtures were well shaken and kept at room temperature in the dark for 30 min. The absorbance was measured at 517 nm using a double beam UV spectrophotometer. Ethanol was used as a negative control; ascorbic acid was used as a standard antioxidant. The radical scavenging activity was calculated as a percentage of DPPH radical discoloration, using the equation:

$$\mathrm{Percentage}\;\mathrm{of}\;\mathrm{DPPH}\;\mathrm{scavenging}\;\mathrm{activity}=\frac{Ao\;\;\;-\;\;Ai}{Ao}\times 100$$

where, Ao is the absorbance of the control and Ai is the absorbance of the test compound.

### 2.5. Lipid Peroxidation Assay

The TBARS were measured as per the method adopted by Tappel and Zalkin [38] with slight modification. Rats (*Rattus norvegicus*) were decapitated and the tissues were removed. Samples were blended for 3 min with 25 mL of 20% TCA. Slurry was kept for 10 min. It was filtered through whatman no. 42 filter paper. Then, 5 mL of TBA reagent was added to 5 mL of sample aliquot (filtrate). After mixing the contents, tubes were kept in a boiling water bath for 35 min. O.D was measured by spectrophotometer at 532 nm. Blank was run simultaneously.

The MDA concentration of the sample was calculated using an extinction coefficient of 1.56 × 10^5^ M^−1^ cm^−1^.

### 2.6. Detection of DNA Strand Breaks

Using the method described by Muiras [39], DNA single-strand breaks were detected by converting double-stranded CCC DNA to double-stranded open circular DNA (OC). Eppendorf polypropylene tubes of 1.5 mL capacity were used for the tests. Next, 0.5 μg of plasmid pBR322 DNA was incubated with increasing concentrations of CdCl_2_ (10–50 µM) and 10 mM H_2_O_2_ at 37 °C for 1 h. Total reaction mixture volume was 30 μL, containing 10 mM Tris HCl (pH 7.5). Then, 0.05% bromophenol blue and 50% (*v*/*v*) glycerol was added as a tracking dye. After incubation, samples were run on 0.9% Agarose gel in 1X TAE buffer in the presence of ethidium bromide (0.25 g/mL) at 90 V for 30 min. At the end, the gel was photographed under UV illuminator. All studies were repeated at least three times under identical conditions.

### 2.7. DNA Protection Assay

The DNA nicking assay, proposed by Lee [40] with some minor modifications, was used to assess the ability of various concentrations of plant extract to protect the pBR322 plasmid DNA from the damaging effects of reactive oxygen species generated via Fenton’s reagent. The 30 μL reaction mixture contained, in the presence of different concentrations of CR extract mixture with other components as indicated in legends at 37 °C for 1 h. after incubation, 0.05% bromophenol blue and 50% (*v*/*v*) glycerol was added as a tracking dye. The samples were run on 0.9% Agarose gel in a 1X TAE buffer in the presence of ethidium bromide (0.25 g/mL) at 90 V for 30 min. At the end of the run, the gel was photographed with a camera under a UV illuminator. All studies were repeated at least three times under identical conditions [22]. 

### 2.8. Antioxidant Enzyme Activity Assay

Protein estimation was carried out for all the homogenates using method described by Lowry [41]; appropriately diluted samples were used for antioxidant activity determination. The activity of Cu, Zn-superoxide dismutase (SOD) was determined from the inhibition of autoxidation of pyrogallol [42] and catalase (CAT) was determined from its ability to convert H_2_O_2_ into H_2_O [43]. Glutathione peroxidase (GPx) was assayed by the method of [44].

### 2.9. Comet Assay

Comet assay was performed under alkaline conditions essentially according to the procedure of Singh [45] with slight modifications. Fully frosted microscopic slides precoated with 0.5% normal melting agarose at about 50 °C (dissolved in Ca^++^ and Mg^++^ free PBS) were used. The slide coated by NMPA and dry 30 μL cells were mixed with 30 μL of 1.5% LMPA to form a cell suspension and pipetted over the first layer and covered immediately by a coverslip. The agarose layer was allowed to solidify by placing the slides on a flat tray and keeping it on ice for 10 min. The coverslips were removed and a third layer of 1.5% LMPA (30 μL) was pipetted with coverslips placed over it and kept on ice for 5 min for proper solidification of the layer. The coverslips were removed and the slides were immersed in cold lysing solution containing 2.5 M NaCl, 100 mM EDTA, 10 mM Tris, pH 10, and 1% Triton X-100 added just prior to use for a minimum of 2–5 h at 4 °C. After lysis, DNA was allowed to unwind for 30 min in an alkaline electrophoretic solution consisting of 300 mM EDTA, 14 M NAOH, pH > 13. Electrophoresis was performed at 4 °C in field strength of 0.7 V/cm and 300 mA current. The slides were then neutralised with cold 0.4 M Tris, pH 7.5, stained with 75 μL Ethidium Bromide (20 μg/mL) and covered with a coverslip. The slides were placed in a humidified chamber to prevent drying of the gel and analysed the same day. Slides were scored using an image analysis system (Komet 5.5, Kinetic Imaging, Liverpool, UK) attached to an Olympus (CX41) fluorescent microscope and a COHU 4910 (equipped with a 510–560 nm excitation and 590 nm barrier filters) integrated CC camera. Comets were scored at 100× magnification. Images from 50 cells (25 from each replicate slide) were analysed. The parameter taken to assess lymphocytes DNA damage was tail length (migration of DNA from the nucleus, μm) and was automatically generated by comet 5.5 image analysis system.

## 3. Results

### 3.1. CR Extract Reduce Cd Toxicity in Albino Rats (Rattus norvegicus)

The cadmium toxicity significantly increased several haematological parameters such as WBC, PLT, PCV, MCV, and MCH while a decrease in the RBC and Hb content was observed in sub-acute groups with an increasing number of days (Table 1). These altered haematological parameters are brought back to normalcy by pre-treatment of groups with CR extract; a non-significant result was observed for the CR group when compared to the control group, showing CR extract itself does not produce any harmful effects. All the values in the table have been shown as means ± SEM and the values are signified at (*p* < 0.01) compared to control. Significant values indicate a significant difference from the control, whereas non-significant values indicate similarity to the control. The non-significant values obtained from the CR extract group and the CR + Cd group indicate that CR pre-treatment prior to cadmium overload significantly protects the kidney against cadmium stress, while it itself does not produce any ill-effects.

### 3.2. CR Extract Showed Antioxidant Effects

**Figure 1:**
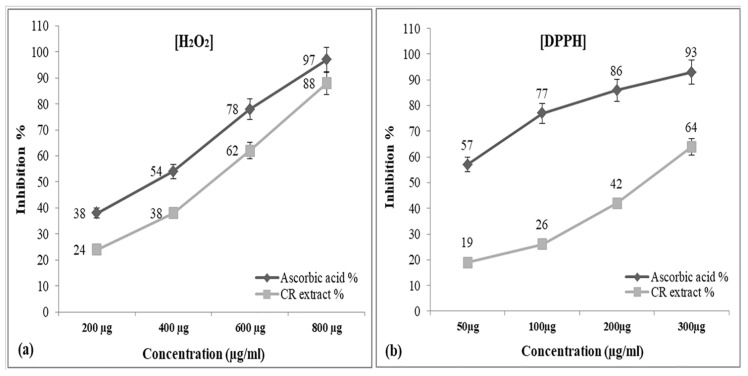
Free Radical inhibition assay of hydroalcoholic extract of CR against standard antioxidant ascorbic acid in the presence of H_2_O_2_ (**a**) and DPPH free radical (**b**). The absorbance of only H_2_O_2_ solution was taken at 230 nm and DPPH free radical scavenging absorbance was taken at 517 nm. Each point denotes the mean ± SEM and value of significance *p* < 0.01 when compared to control.

Next to check whether the CR extract has any antioxidant effects, we performed an H_2_O_2_ scavenging assay. H_2_O_2,_ is a weak oxidising agent that inactivates various enzymes directly, generally by oxidation of thiol (– SH) groups, which can enter cell membranes rapidly. Once it is inside the cell, Cu^+2^ and Fe^+2^ ions react with peroxide, generating hydroxyl radicals, which may be a major reason for the origin of numerous toxic effects [46]. Thus, the removal of hydrogen peroxide as well as oxygen becomes inevitable for the protection of biological systems from oxidative damage. The scavenging efficiency of the hydro-alcoholic CR extract at increasing concentrations (200, 400, 600, and 800 μg/mL) of hydrogen peroxide was denoted as percentage inhibition and compared with standard ascorbic acid. As evident from the inhibition graph, the CR extract was also able to scavenge hydrogen peroxide radical significantly as compared to standard ascorbic acid (Figure 1a). 

The study of DPPH activity assay plays a prominent role for screening plant extract, since samples can be adapted in a short period of time and are sensitive even at remarkably low concentrations of active ingredients [47]. In the assay, DPPH behaves as a free radical which reacts with antioxidants. In turn, the antioxidant reduces DPPH to DPPH_2_ due to electron transfer. As a result, the absorbance decreases. The degree of discoloration indicates the scavenging potential of the antioxidant compounds in terms of hydrogen donating ability. Therefore, to further confirm the antioxidant activities of CR extract, we performed a DPPH radical scavenging assay. The antioxidant capacities of CR extract and Ascorbic acid at different concentrations (50, 100, 200 and 300 μg/mL) showed a gradual dose-dependent increase in the percentage inhibition of free radical formation with the increase in the concentration of the test and standard (Ascorbic Acid) compound (Figure 1b). It is evident from the graph that CR showed a significant amount of DPPH radical scavenging effects compared to the standard. Previously, most studies have reported a positive relation between phenolics, Terpenoid Indole Alkaloids (TIAs), flavonoids content and DPPH radical scavenging activity of plant extracts [48,49].

### 3.3. CR Extract Protects against Lipid Peroxidation of Rat Tissue

**Figure 2:**
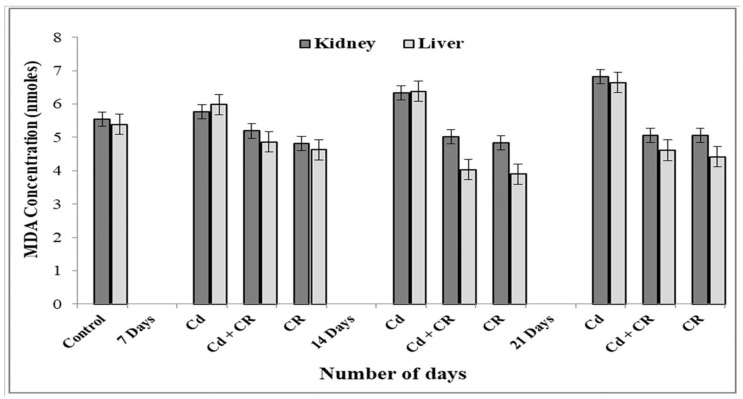
Effect of cadmium and CR extract on lipid peroxidation in rat liver and kidney with increasing number of days, as determined by the concentrations of malondialdehyde (MDA), absorbance was measured at 532 nm and MDA concentration was recorded in nmoles for Cd, CR extract, Cd + CR extract, each column shows the mean ± SEM and value of significance *p* < 0.01 when compared to control.

MDA concentration, an end product of lipid peroxidation, is a classical marker of oxidative stress in cells. Therefore, to check whether CR extract can provide protection against lipid peroxidation, we performed a lipid peroxidation assay. We have observed that treatment with Cd resulted in a 23% increase in overall lipid peroxidation as compared to control in both kidney and liver. As evident from (Figure 2), the Cd group were given a dose of CR over a period of 7 to 21 days. As a result, there was a considerable reduction in MDA content, with up to 35% protection against lipid peroxidation in the kidneys and 44% protection in the liver. This demonstrates that active ingredients present in CR extract play a major role in decreasing the rate of lipid peroxidation in the membrane caused by Cd toxicity. 

### 3.4. CR Extract Showed Protection against Cd Induced Oxidative DNA Damage

**Figure 3:**
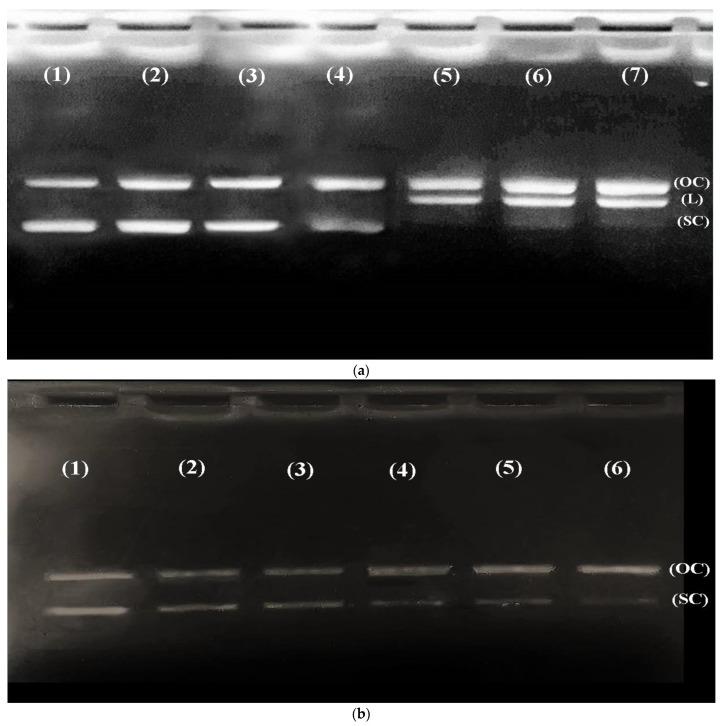

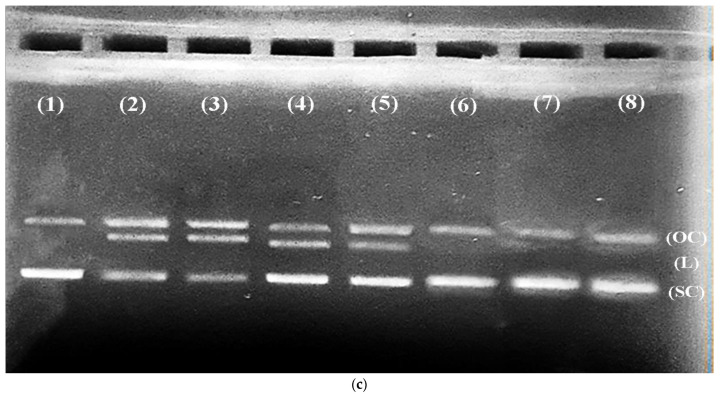
(**a**): Agarose gel electrophoretic pattern of EtBr stained pBR322 Plasmid DNA after treatment with Cadmium in presence and absence of H_2_O_2_. The reaction mixture (30 µL) contained 0.5 µg PBR322 plasmid DNA; 10 mM Tris HCl (pH7.5) indicated concentration of Cadmium and hydrogen peroxide. Incubation of the sample was carried out at 37 °C for 30 min. Lane 1: DNA alone; Lane 2: DNA + Cadmium 20 µM; Lane 3: DNA + H_2_O_2_ 20 mM; Lane 4: DNA + Cadmium 10 µM + H_2_O_2_ 10 mM; Lane 5: DNA + Cadmium 20 µM + H_2_O_2_ 10 mM; Lane 6: DNA + Cadmium 30 µM + H_2_O_2_ 10 mM; Lane 7: DNA + Cadmium 50 µM + H_2_O_2_ 10 mM. (**b**): Each well contained sample as follows; Lane 1: DNA alone; Lane 2: DNA + cadmium 10 µM + H_2_O_2_ 1 mM; Lane 3: DNA + cadmium 10 µM + H_2_O_2_ 10 mM; Lane 4: DNA + cadmium 10 µM + H_2_O_2_ 20 mM; Lane 5: DNA + cadmium 10 µM + H_2_O_2_ 30 mM; Lane 6: DNA + cadmium 10 µM + H_2_O_2_ 50 mM. (**c**): EtBr stained pBR322 Plasmid DNA after treatment with Cadmium with H_2_O_2_ in the presence of the CR extract. All the reaction conditions were maintained as above with indicated concentration of cadmium and hydrogen peroxide with increasing concentration of CR extract. Lane 1: DNA alone; Lane 2: DNA + cadmium 20 µM + H_2_O_2_ 20 mM + extract 5 µg/mL; Lane 3: DNA + cadmium 20 µM + H_2_O_2_ 20 mM + extract 10 µg/mL; Lane 4: DNA + cadmium 20 µM + H_2_O_2_ 20 mM + extract 20 µg/mL; Lane 5: DNA + cadmium 20 µM + H_2_O_2_ 20mM + extract 30 µg/mL; Lane 6: DNA + cadmium 20 µM + H_2_O_2_ 20 mM + extract 50 µg/mL; Lane 7: DNA + cadmium 20 µM + H_2_O_2_ 20 mM + extract 75 µg/mL; Lane 8: DNA + cadmium 20 µM + H_2_O_2_ 20 mM + extract 100 µg/mL.

Reactive oxygen species generated by Fenton-like reactions are known to cause oxidatively-induced breaks in DNA strands to yield their open, circular or relaxed forms. Exposure of plasmid DNA to Fenton’s reagent ultimately results in strand breaks, mainly due to the generation of ROS and the subsequent free radical-induced reaction on plasmid DNA. Reactive oxygen species react with the nitrogenous bases of DNA producing base radicals and sugar radicals. However, base radicals in turn react with the sugar moiety, causing breakage of the sugar phosphate backbone of DNA, resulting in a DNA strand breaking [50]. It was reported that hydroxyl radicals are also produced from H_2_O_2_ through Fenton-like reactions [39]. Thus, in order to evaluate whether cadmium is capable of inducing oxidative DNA damage, an in vitro experimental strategy was designed where pBR322 plasmid DNA was exposed to various concentrations of Cd^+2^ and H_2_O_2_. Therefore, 0.5 μg of pBR322 plasmid DNA was incubated with increasing concentrations (10, 20, 30 50 μM) of CdCl_2_ in the presence of H_2_O_2_ for 1 h at 37 °C, showing a significant dose-dependent increase in plasmid DNA breakage, resulting in the conversion of DNA from the CCC form to an open circular (OC) and linear (L) form (Figure 3a). Thus, it is evident that more reactive oxygen species were produced in the presence of hydrogen peroxide in the presence of an increasing concentration of CdCl_2,_ causing single and double-stranded DNA breaks, showing Cd as an effector molecule in this reaction. However, there seems to be a very slight change in the SC structures on increasing hydrogen peroxide concentration in the presence of CdCl_2_ (Figure 3b).

Next, to check the DNA protection capabilities of the CR extract, we performed a DNA protection assay. Figure 3c shows the results for DNA protection, performed in the presence of increasing concentrations (5 µg/mL, 10 µg/mL, 20 µg/mL, 30 µg/mL, 50 µg/mL, 75 µg/mL, and 100 µg/mL) of CR extract. A dose-dependent increase in the formation of SC was observed, which suggests that the CR extract can protect the DNA from damage caused by oxidative stress. 

### 3.5. Cd Causes Oxidative Stress Independent of Copper 

**Figure 4:**
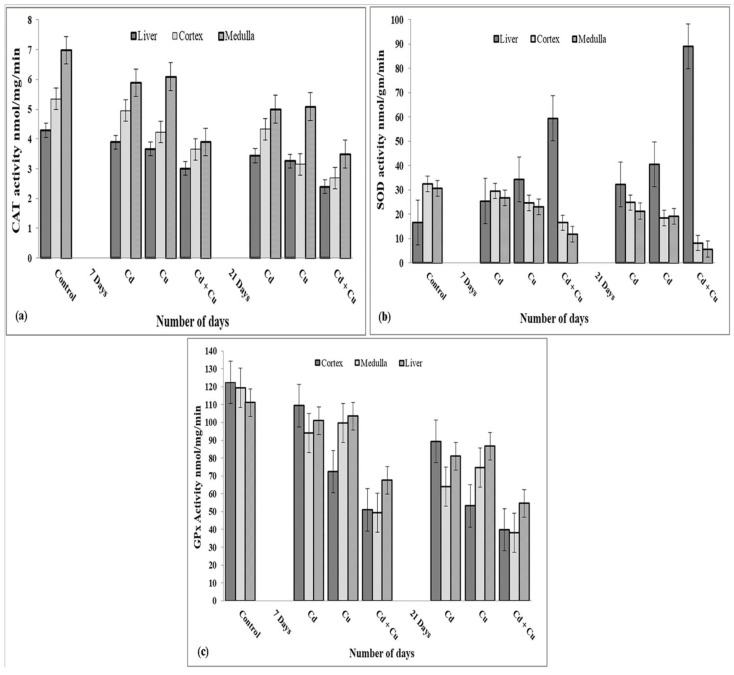
Effect on antioxidant enzymes activities of treated groups. Represented graphs for the time course of the three-enzyme assay. Activity of CAT (**a**), SOD (**b**) and GPx (**c**) in the presence of Cu, Cd and Cu + Cd was tested for 7 and 21 days (treated groups) compared to untreated control. Each column shows the mean ± SEM and value of significance *p* < 0.01 when compared to the control.

Copper (Cu) is an abundant trace metal ion found in all living cells in the oxidised Cu(II) and reduced Cu(I) states. It is an important catalytic cofactor in redox chemistry and is required by many proteins that carry out fundamental biological functions [51,52]. The role of copper has been reported to cause oxidative stress. Therefore, to rule out the possibility that the oxidative stress caused by Cd is dependent on Cu, we have studied the role of Cu along with Cd in different scenarios and checked the activities of antioxidant enzymes in the presence of three metal-treated groups (Cd, Cu, and Cd + Cu) for an increasing number of days. In the livers of Cd, Cu, and Cd + Cu treated rats, 19.1%, 21%, and 40% reductions in catalase activity were observed, respectively. A similar pattern of catalase activity was observed in kidneys’ of Cd, Cu, Cd + Cu treated rats (Cortex: Cd, 21.01%; Cu, 40.08%; Cd + Cu, 49.18% and Medulla: Cd, 26.78%; Cu, 24.53%; Cd + Cu, 48.57%), Figure 4a. The SOD activity showed a contrary pattern depending on the organ assayed. We observed an increase in the overall SOD activity in the liver while comparing the metal-treated group with control values (Cd, 98.69%; Cu, 148.3%; Cd + Cu, 452.5%). Possible reasons for the enhanced activity of SOD in the liver may be due to the compensatory mechanism for the conversion of more damaging ROS to the less damaging H_2_O_2_ and glutathione conjugates [53,54], In contrast to the liver, SOD activity was evidently reduced in the kidneys of the treated rats (Cortex: Cd, 20.8%; Cu, 42.71%; Cd + Cu, 75.21%; and Medulla: Cd, 29.5%; Cu, 37.7%; Cd + Cu, 81.7%) Figure 4b. Glutathione peroxidase activity was also reduced significantly in the liver (Cd, 26.8%; Cu, 21.8%; Cd + Cu, 50.6%) and kidney (Cortex: Cd, 25.7%; Cu, 60.9%; Cd + Cu, 62.8% and Medulla: Cd, 46.4%; Cu, 37.2%; Cd + Cu, 67.3%) in comparison to the control, as shown in Figure 4c. In a broader perspective, the enzymatic alterations were slightly higher in Cu-treated rats when compared with Cd-treated groups. Moreover, alterations due to the combination of Cd and Cu were specifically additive in nature, showing a higher degree of damage to antioxidant enzymes compared to individually treated groups. 

In the liver and kidney, concentrations of antioxidant enzymes such as CAT, GPx, and SOD were significantly reduced upon treatment with cadmium as compared to the control group, while CR + Cd rescued antioxidant enzymes when compared to the Cd-treated group (Table 2, Table 3 and Table 4). It is well known that oxidation of biological substrates leads to various diseases [55]. Antioxidants, flavonoids, alkaloids, and phytochemicals from CR are now being used to counteract oxidation at the biological level, which is a safer approach than using pharmaceutical drugs to reduce oxidation [56]. The plant investigated is used in traditional medicine for various medicinal and pharmacological effects. Furthermore, our study shows that CR exhibits good antioxidant activity in vivo.

### 3.6. CR Extract Protects against Cellular DNA Damage Caused by Cd in Rat Tissues

**Figure 5:**
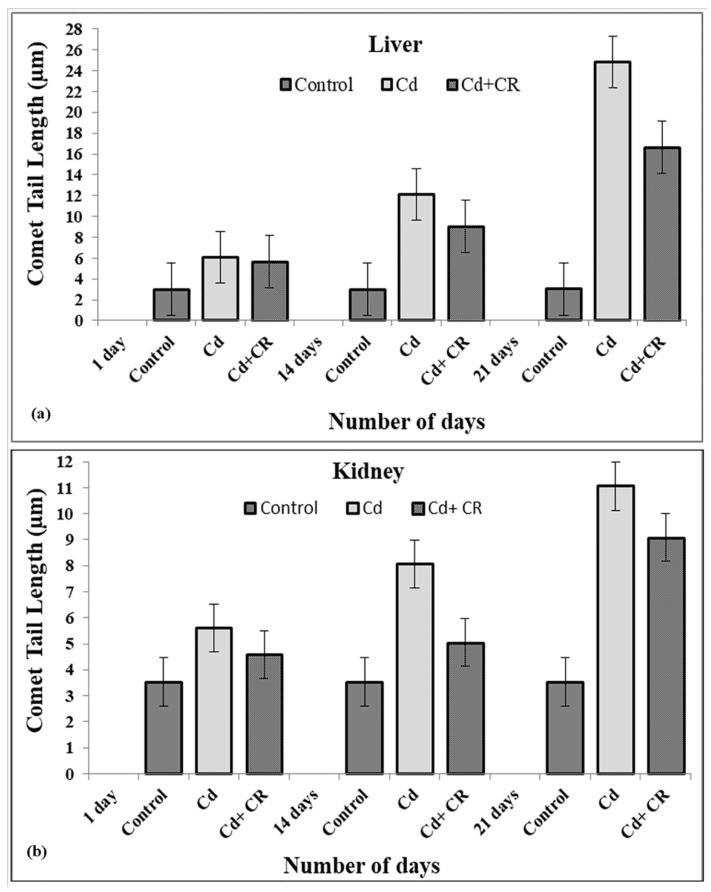
Comet assay-DNA tail length formations of Liver cells (**a**) and Kidney cells (**b**) of rats exposed to cadmium and CR extract doses for 21 days. Comet tail length (µm) plotted against number of days as a parameter of DNA breakage in presence of 8.8 mg/kg body weight and 500 mg/kg body weight CR extract. Each column represents the mean ± SEM of three independent experiments, value of significance *p* < 0.05 when compared to the control.

As shown earlier, Cd, being a toxic metal ion, causes DNA breakage, which was significantly decreased in the presence of CR extract. Therefore, for further validation, we have tested the effects of Cd and CR extract in treated groups of kidney and liver of rat tissues in a slightly modified comet assay. Figure 5a,b clearly indicate that in all Cd-treated groups (8.8 mg/kg body weight, for 1, 14, and 21 days), there is a gradual increase in cellular DNA breakage with an increasing number of days compared to the control, depicted by enhanced tail formation. On the other hand, the extent of DNA breakage seems to significantly decrease in response to CR extract in treated groups. These results clearly indicate the protective role of CR extract against Cd-induced cellular DNA breakage in the liver and kidney. 

## 4. Discussion

We have recently demonstrated the Cd-induced stress parameters and their damaging effects on rats’ kidneys and their bioremediation by CR extract [33]. The present study, however, deals with the molecular aspect of Cd-induced toxicity in cells and demonstrates the significant role of Cd as an effector molecule, while significant protection against toxicity is shown by CR extract in the process. First, to assess the detrimental effects of Cd in living cells, rats were fed with an 8.8 mg/kg dose of Cd over a period of 21 days, and further studies were carried out on the harvested kidney and liver tissues. Haematological parameters show a dose-dependent decrease in the total Hb content with an increase in the MCV and MCH values (Table 1). Mostly, MCV and MCH values are often consistent with the Hb value. However, our results show low Hb and high MCH values, which may be due to the folate deficiency caused by heavy metal toxicity. These results clearly show that overall RBC count is decreased, which may be due to the decreased concentration of folate, as folate is important for making RBCs and thus affects the overall Hb content under stress conditions [57,58]. Furthermore, these results are consistent with the increase in lipid peroxidation in the presence of Cd (Figure 2). In this context, we should not forget that as lipid peroxidation increases, RBCs tend to release more and more Hb due to the formation of MDA-TBA adducts in the membrane [59,60]. This may be a leading cause of haemolytic anaemia, which may result in increased haemoglobin breakdown, causing it to release Fe that reacts with H_2_O_2_ to form hydroxyl-like species [61]. To further confirm the fact that Cd exerts oxidative stress, which may lead to DNA breakage, we studied the effects of Cd in the presence and absence of H_2_O_2_, on pBR322 plasmid DNA. As reports suggest, Cd alone is unable to cause any DNA breakage in the plasmid DNA [20]. However, we observed that Cd in the presence of H_2_O_2_ was able to change the SC structure of plasmid to OC and linear forms and no such breakage was observed in the presence of H_2_O_2_ alone (Figure 3a) [62]. DNA breakage was significantly enhanced when Cd was incubated with DNA at an increasing concentration and H_2_O_2_ was held constant. On the other hand, the degree of such DNA breakage was not found to be very significant when Cd was supplemented in the presence of gradually increasing H_2_O_2_ concentration (Figure 3b). Since, Cd does not belong to a redox-active or transition metal ion group, it may not directly cause the generation of ROS in the system but may trigger an assembly of Fenton-type reactions in the living system, leading to the generation of ROS, which may cause DNA breakage by exerting oxidative stress [20,63]. In order to prevent this oxidative DNA breakage, the ability of CR extract was demonstrated in Figure 3c, which clearly showed the gradual dose-dependent conversion of OC and linear forms back to the native form. These results are in consonance with the results presented in Figure 1a,b, which showed a significant increase in the scavenging activity of the extract in a dose dependent-manner when compared to the standard. The DNA damaging and protective ability of Cd and CR extract, respectively, is also confirmed by the results obtained from the comet assay of rat liver and kidney tissues (Figure 5a,b). The comet assay showed increasing cellular DNA breakage with an increasing number of days in the presence of Cd, while the degree of such DNA breakage was decreased when tissues were subjected to CR extract, showing the protective role of CR.

Since copper is an important redox metal ion found in the cell, which is required to carry out various biological functions, there is a possibility that it may increase the generation of ROS under stress conditions and act as one of the main effector metal ions and may affect the role of Cd in ROS generation [64]. Thus, to assess the role of copper in this process, various antioxidant enzyme activities were tested in the presence and absence of Cu and Cd. The results obtained in (Figure 4a–c) showed an additive effect in the overall decrease in AO enzyme activity in kidney cells. Similar results were obtained in the liver, except for SOD concentration, which was observed to be increased ⸟4.5 folds, which may be due to a compensatory mechanism for the conversion of more damaging ROS to the less damaging H_2_O_2_ and glutathione conjugates [53,54]. The bioremediation of AO enzymes by CR extract can be clearly seen in (Table 2, Table 3 and Table 4), where AO enzyme concentrations were increased when compared to Cd-treated groups. 

## 5. Conclusions

The findings that are given here show the excellent antioxidant potential of CR extract, as confirmed by the H_2_O_2_/DPPH free radical scavenging assay, which significantly protected cells against oxidative membrane damage caused by Cd toxicity, which was confirmed by the decrease in MDA concentration. It is important to note that Cd ions are able of promoting significant ROS formation in the presence of H_2_O_2_ in a dose-dependent manner. This is not the case in the absence of H_2_O_2_ or with rising concentrations of H_2_O_2_. This will trigger the disruption of a variety of different intracellular activities, as well as induce ROS-mediated oxidative/cellular DNA breakage in addition to enhanced lipid peroxidation, which will ultimately result in cell death. The toxicity that was caused by the presence of Cd was significantly reduced in the cells that were treated with the CR extract, which includes a number of phyto-ingredients that are known to be active. By demonstrating its potential role in phytoremediation against such toxins, it is implied that the use of CR-based extracts might have some biological significance and could be a topic of interest for future research directions.

## Figures and Tables

**Table 1:** The different haematological parameter of the experimental groups and that of the negative control, WBC, White blood cells (×10^3^/μL); RBC, red blood cells (×10^6^/μL); PLT, platelets (×10^3^/μL); PCV, packed cell volume (%); MCH, mean corpuscular haemoglobin (pg); MCV, mean corpuscular volume (fL); All the values in the table has been shown as means ± SEM. *p* < 0.01, when compared to control. All values show significant and non-significant difference when compared to control (* = significant; ns = Non-significant).

7 days	**Parameters**	**Control**	**Cadmium**	**Cadmium + CR**	**CR**
	RBC	8.42 ± 3.19	5.87 ± 2.23 *	6.98 ± 2.64 ^ns^	8.61 ± 3.26 ^ns^
	WBC	12.26 ± 0.001	14.98 ± 0.153 *	12.98 ± 0.053 ^ns^	12.07 ± 0.022 ^ns^
	PLT	625.25 ± 15.58	961.05 ± 156.49 *	612.04 ± 14.89 *	621.78± 13.45 ^ns^
	Hb	158.01 ± 7.58	115.98 ± 6.08 *	148.54 ± 6.98 *	155.06 ± 7.35 ^ns^
	PCV	42.05 ± 0.81	45.05 ± 0.02 *	41.91 ± 0.66 ^ns^	42.01 ± 0.54 ^ns^
	MCV	59.29 ± 1.78	62.01 ± 2.45 *	58.97 ± 1.49 *	58.79 ± 1.66 ^ns^
	MCH	18.79 ± 0.56	19.17 ± 1.45 *	18.69 ± 0.43 ^ns^	16.28 ± 0.73 ^ns^
21 days	RBC	8.73 ± 3.30	5.67 ± 2.14 *	7.25 ± 2.74 ^ns^	8.58 ± 3.25 ^ns^
	WBC	11.77 ± 0.009	13.78 ± 1.29 *	12.59 ± 0.034 ^ns^	11.98 ± 0.01 ^ns^
	PLT	624.29 ± 14.88	923.50 ± 186.66 *	619.14 ± 13.99 ^ns^	621.68 ± 12.45 ^ns^
	Hb	156.98 ± 7.56	118.02 ± 6.57 *	157.08 ± 7.54 ^ns^	142.58 ± 6.57 ^ns^
	PCV	43.03 ± 089	44.58 ± 0.03 *	42.88 ± 0.65 ^ns^	42.98 ± 0.52 ^ns^
	MCV	60.01 ± 1.97	63.47 ± 0.71 *	60.96 ± 1.73 ^ns^	60.00 ± 1.49 ^ns^
	MCH	18.49 ± 1.24	22.03 ± 0.66 *	19.46 ± 1.78 *	18.20 ± 1.59 ^ns^

**Table 2:** GPx (IU/L) of *Rattus norvegicus* after acute and sub-acute treatment with CR followed by cadmium chloride, All the values in the table has been shown as means ± SEM. *p* < 0.01, when compared to control. All values show significant and non-significant difference when compared to control (* = significant; ns = Non-significant).

Liver		**Groups**	**Control**	**Cadmium Treated**	**Cadmium + CR**	**CR**
	**Sets**					
	Set I: Acute (1d)		5.23 ± 0.003	3.40 ± 0.006 *	5.347 ± 0.003 *	4.18 ± 0.008 ^ns^
	Set II: Sub-acute (7ds)		5.31 ± 0.004	3.03 ± 0.001 *	5.38 ± 0.007 *	5.22 ± 0.008 ^ns^
	Set III: Sub-acute (14ds)		5.29 ± 0.005	3.02 ± 0.003 *	5.35 ± 0.004 *	4.98 ± 0.007 ^ns^
	Set IV: Sub-acute (21ds)		5.38 ± 0.003	2.66 ± 0.006 *	5.37 ± 0.004 ^ns^	5.42 ± 0.006 ^ns^
Kidney	Set I: Acute (1d)		6.42 ± 0.003	4.58 ± 0.006 *	6.47 ± 0.003 *	5.88 ± 0.008 ^ns^
	Set II: Sub-acute (7ds)		6.53 ± 0.004	4.08 ± 0.001 *	6.68 ± 0.007 *	6.49 ± 0.008 ^ns^
	Set III: Sub-acute (14ds)		6.41 ± 0.005	4.09 ± 0.003 *	6.40 ± 0.004 ^ns^	6.27± 0.007 ^ns^
	Set IV: Sub-acute (21ds)		6.57 ± 0.003	3.71 ± 0.006 *	6.56 ± 0.004 ^ns^	6.25 ± 0.006 ^ns^

**Table 3:** SOD (IU/L) of *Rattus norvegicus* after acute and sub-acute treatment with CR followed by cadmium chloride, All the values in the table has been shown as means ± SEM. *p* < 0.01, when compared to control. All values show significant and non-significant difference when compared to control (* = significant; ns = Non-significant).

Liver		**Groups**	**Control**	**Cadmium Treated**	**Cadmium + CR**	**CR**
	**Sets**					
	Set I: Acute (1d)		9.04 ± 0.004	4.82 ± 0.024 *	10.01 ± 0.003 *	7.85 ± 0.004 ^ns^
	Set II: Sub-acute (7ds)		9.13 ± 0.005	4.24 ± 0.010 *	9.80 ± 0.078 *	7.06 ± 0.012 ^ns^
	Set III: Sub-acute (14ds)		9.11 ± 0.004	4.05 ± 0.003 *	10.13 ± 0.093 *	8.98 ± 0.007 ^ns^
	Set IV: Sub-acute (21ds)		9.25 ± 0.009	3.96 ± 0.006 *	9.94 ± 0.066 *	9.03 ± 0.006 ^ns^
Kidney	Set I: Acute (1d)		16.01 ± 0.004	10.79 ± 0.004 *	18.29 ± 0.003 *	9.35 ± 0.004 ^ns^
	Set II: Sub-acute (7ds)		16.13 ± 0.005	11.09 ± 0.003 *	14.59 ± 0.018 *	10.46 ± 0.002 ^ns^
	Set III: Sub-acute (14ds)		16.51 ± 0.004	9.09 ± 0.003 *	12.13 ± 0.023 *	11.98 ± 0.007 ^ns^
	Set IV: Sub-acute (21ds)		16.51 ± 0.009	8.86 ± 0.006 *	13.94± 0.046 *	15.93 ± 0.003 ^ns^

**Table 4:** Catalase (IU/L) of *Rattus norvegicus* after acute and sub-acute treatment with CR followed by cadmium chloride, All the values in the table has been shown as means ± SEM. *p* < 0.01, when compared to control. All values show significant and non-significant difference when compared to control (* = significant; ns = Non-significant).

Liver		**Groups**	**Control**	**Cadmium Treated**	**Cadmium + CR**	**CR**
	**Sets**					
	Set I: Acute (1d)		42.14 ± 0.004	24.82 ± 0.024 *	43.01 ± 0.003 *	37.26 ± 0.004 ^ns^
	Set II: Sub-acute (7ds)		42.17 ± 0.005	24.24 ± 0.010 *	41.60 ± 0.078 *	37.36 ± 0.012 ^ns^
	Set III: Sub-acute (14ds)		42.15 ± 0.004	24.05 ± 0.003 *	40.13 ± 0.093 *	38.98 ± 0.004 ^ns^
	Set IV: Sub-acute (21ds)		43.55 ± 0.009	23.96 ± 0.002 *	39.94 ± 0.006 *	39.23 ± 0.003 ^ns^
Kidney	Set I: Acute (1d)		52.43 ± 0.010	34.31 ± 0.002 *	53.24 ± 0.103 *	39.56 ± 0.005 ^ns^
	Set II: Sub-acute (7ds)		52 ± 0.004	31.78 ± 0.002 *	52.68 ± 0.003 *	41.98 ± 0.002 ^ns^
	Set III: Sub-acute (14ds)		51.88 ± 0.004	29.66 ± 0.003 *	52.28 ± 0.007 *	49.36 ± 0.003 ^ns^
	Set IV: Sub-acute (21ds)		52.30 ± 0.042	27.03 ± 0.014 *	53.17 ± 0.005 *	51.96 ± 0.014 ^ns^

## Data Availability

The data presented in this study are available in article.

## Abbreviations

Catharanthus roseus, CR; Antioxidant, AO; Malondialdehyde, MDA; Diphenyl picrylhydrazyl, DPPH; Cadmium, Cd; Copper, Cu; Plasmid bolivar and Rodrigues, pBR322; Catalase, CAT; Superoxide dismutase, SOD; Glutathione peroxidase, GPx; Hydrogen peroxide, H_2_O_2_; Open circle OC; Linear, L; Supercoiled, SC.

## References

[1] UNEP, GPA (2006). The State of the Marine Environment: Trends and Processes.

[2] Bradl H. (2002). Heavy Metals in the Environment: Origin, Interaction and Remediation.

[3] He Z.L., Yang X.E., Stoffella P.J. (2005). Trace elements in agroecosystems and impacts on the environment. J. Trace Elem. Med. Biol..

[4] Lv B., Zhuo J.Z., Peng Y.D., Wang Z. (2022). Comparative analysis of cadmium-induced toxicity and survival responses in the wolf spider *Pirata subpiraticus* under low-temperature treatment. Environ. Sci. Pollut. Res. Int..

[5] Borowska S., Brzóska M.M. (2015). Metals in cosmetics: Implications for human health. J. Appl. Toxicol..

[6] World Health Organization (2019). Preventing Disease through Healthy Environments: Exposure to Cadmium: A Major Public Health Concern. https://apps.who.int/iris/handle/10665/329480.

[7] Johnston P., Santillo D., Stringer R., Ashton J., McKay B., Verbeek M., Jackson E., Landman J., van den Broek J., Samsom D. (1998). Report on the World’s Oceans.

[8] World Health Organization (2021). The Public Health Impact of Chemicals: Knowns and Unknowns: Data Addendum for 2019. https://apps.who.int/iris/handle/10665/342273.

[9] Ijaz M.U., Batool M., Batool A., Al-Ghanimd K.A., Zafar S., Ashraf A., Al-Misned F., Ahmed Z., Shahzadi S., Samad A. (2021). Protective effects of vitexin on cadmium-induced renal toxicity in rats. SJBS.

[10] Faroon O., Ashizawa A., Wright S., Tucker P., Jenkins K., Ingerman L., Rudisill C. (2012). Chapter 3: Health effects. Toxicological Profile for Cadmium.

[11] Atef M.M.A., Ibrahim A.A.F., Abd EL-Latif A.N., Aziz W.S. (2014). Antioxidant effects of curcumin against cadmium chloride-induced oxidative stress in the blood of rats. J. Pharmacol. Phytother..

[12] World Health Organization & International Programme on Chemical Safety (1992). Cadmium: Environmental Aspects/Published under the Joint Sponsorship of the United Nations Environment Programme, The International Labour Organisation, and the World Health Organization. https://apps.who.int/iris/handle/10665/39366.

[13] Takiguchi M., Yoshihara S. (2006). New aspects of cadmium as endocrine disruptor. Environ. Sci..

[14] Siu E.R., Mruk D.D., Porto C.S., Cheng C.Y. (2009). Cadmium-induced testicular injury. Toxicol. Appl. Pharmacol..

[15] Jin T., Lu J., Nordberg M. (1998). Toxicokinetics and biochemistry of cadmium with special emphasis on the role of metallothionein. Neurotoxicology.

[16] Meplan C., Mann K., Hainaut P. (1999). Cadmium Induces Conformational Modifications of Wild-type p53 and Suppresses p53 Response to DNA Damage in Cultured Cells. J. Biol. Chem..

[17] Zhong Z.J., Troll W., Koenig K.L., Frenkel K. (1990). Carcinogenic sulfide salts of nickel and cadmium induce H_2_O_2_ formation by human polymorphonuclear leukocytes. Cancer Res..

[18] Rafati R.M., Rafati R.M., Kazemi S., Moghadamnia A.A. (2017). Cadmium toxicity and treatment: An update. Caspian J. Intern. Med..

[19] Stohs S.J., Bagchi D., Hassoun E., Bagchi M. (2001). Oxidative mechanisms in the toxicity of chromium and cadmium ions. J. Environ. Pathol. Toxicol. Oncol..

[20] Badisa V.L., Latinwo L.M., Odewumi C.O., Ikediobi C.O., Badisa R.B., Ayuk-Takem L.T., Nwoga J., West J. (2007). Mechanism of DNA damage by cadmium and interplay of antioxidant enzymes and agents. Environ. Toxicol..

[21] Daniel R.L., Phillips D.H. (1999). Oxidative DNA damage mediated by copper (II), iron (II) and nickel (II) Fenton reactions: Evidence for site-specific mechanisms in the formation of double-strand breaks, 8-hydroxydeoxyguanosine and putative intra-strand cross-links. Mutat. Res. Fundam. Mol. Mech. Mutagen..

[22] Arif H., Sohail A., Farhan M., Rehman A.A., Ahmad A., Hadi S.M. (2018). Flavonoids-induced redox cycling of copper ions leads to generation of reactive oxygen species: A potential role in cancer chemoprevention. Int. J. Biol. Macromol..

[23] Illán-Cabeza N.A., Vilaplana R.A., Alvarez Y., Akdi K., Kamah S., Hueso-Ureña F., Quirós M., González-Vílchez F., Moreno-Carretero M.N. (2005). Synthesis, structure and biological activity of a new and efficient Cd (II)-uracil derivative complex system for cleavage of DNA. J. Biol. Inorg. Chem..

[24] Natarajan A., Ahmed K.S.Z., Sundaresan S., Sivaraj A., Devi K., Kumar B.S. (2012). Effect of aqueous flower extract of *catharanthus roseus* on alloxan induced diabetes in male albino rats. Int. J. Pharm. Sci. Drug Res..

[25] Saboon C.S.K., Arshad S., Amjad M.S., Akhtar M.S., Akhtar M., Swamy M., Sinniah U. (2019). Natural Compounds Extracted from Medicinal Plants and Their Applications. Natural Bio-Active Compounds.

[26] Van-Der-Heijden R., Jacobs D.I., Snoeijer W., Hallard D., Verpoorte R. (2004). The Catharanthus alkaloids: Pharmacognosy and biotechnology. Curr. Med. Chem..

[27] Chen Q., Lu X., Guo X., Guo Q., Li D. (2017). Metabolomics characterization of two Apocynaceae plants, *Catharanthus roseus* and *Vinca minor*, using GC-MS and LC-MS methods in combination. Molecules.

[28] Chaturvedi V., Goyal S., Mukim M., Meghani M., Patwekar F., Patwekar M., Khan S.K., Sharma G.N. (2022). A Comprehensive Review on *Catharanthus roseus* L. (G.) Don: Clinical Pharmacology, Ethnopharmacology and Phytochemistry. J. Pharmacol. Res. Dev..

[29] Mustafa N.R., Verpoorte R. (2007). Phenolic compounds in *Catharanthus roseus*. Phytochem Rev..

[30] Levêque D., Jehl F. (2007). Molecular pharmacokinetics of *catharanthus* (vinca) alkaloids. J. Clin. Pharmacol..

[31] Arif H., Rehmani N., Farhan M., Ahmad A., Hadi S.M. (2015). Mobilization of Copper ions by Flavonoids in Human Peripheral Lymphocytes Leads to Oxidative DNA Breakage: A Structure Activity Study. Int. J. Mol. Sci..

[32] Panchal H., Sachdeva S.N., Bhardwaj J.K. (2022). Ultrastructural analysis of cadmium-induced toxicity and its alleviation by antioxidant quercetin in caprine testicular germ cells in vitro. Ultrastruct. Pathol..

[33] Hashim M., Tabassum B., Abd Allah E.F., Hashem A., Bajaj P. (2018). Bioremediation of cadmium induced renal toxicity in *Rattus norvegicus* by medicinal plant *Catharanthus roseus*. Saudi J. Biol. Sci..

[34] Golla U.R., Gajam P.K., Mohammad A.R., Kumar K.A., Raj B.S.S. (2011). Assessment of bioactivity of *Desmostachya bipinnata* (L.) Stapf using brine shrimp (*artemia salina*) lethality assay. Pharmacologyonline.

[35] Brunning R.D. (1995). Hematology: Basic Principles and Practice. JAMA J. Am. Med. Assoc..

[36] Gülçin I., Alici H.A., Cesur M. (2005). Determination of in vitro antioxidant and radical scavenging activities of propofol. Chem. Pharm. Bull..

[37] Abe N., Murata T., Hirota A. (1998). Novel DPPH Radical Scavengers, Bisorbicillinol and Demethyltrichodimerol, from a Fungus. Biosci. Biotechnol Biochem..

[38] Tappel A.L., Zalkin H. (1959). Inhibition of lipid peroxidation in mitochondria by vitamin E. Arch. Biochem. Biophys..

[39] Muiras M.L., Giacomoni P.U., Tachon P. (1993). Modulation of DNA breakage induced via the Fenton reaction. Mutat. Res..

[40] Lee J.C., Kim H.R., Kim J., Jang Y.S. (2002). Antioxidant property of an ethanol extract of the stem of *Opuntia ficus-indica* var. saboten. J. Agric. Food Chem..

[41] Lowry O.H., Rosebrough N.J., Farr A.L., Randall R.J. (1951). Protein measurement with the Folin phenol reagent. J. Biol. Chem..

[42] Marklund S., Marklund G. (1974). Involvement of the superoxide anion radical in the autoxidation of pyrogallol and a convenient assay for superoxide dismutase. Eur. J. Biochem..

[43] Aebi H. (1984). Catalase in vitro. Methods Enzymol..

[44] Flohé L., Günzler W.A. (1984). Assays of glutathione peroxidase. Methods Enzymol..

[45] Singh N.P., McCoy M.T., Tice R.R., Schneider E.L. (1988). A simple technique for quantitation of low levels of DNA damage in individual cells. Exp. Cell Res..

[46] Miller M.J., Sadowska-Krowicka H., Chotinaruemol S., Kakkis J.L., Clark D.A. (1993). Amelioration of chronic ileitis by nitric oxide synthase inhibition. J. Pharmacol. Exp. Ther..

[47] Sanchez-Moreno C. (2002). Methods used to evaluate the free radical scavenging activity in foods and biological systems. Food Sci. Technol. Int..

[48] Shahidi F., Wanasundara P.K. (1992). Phenolic antioxidants. Crit. Rev. Food Sci. Nutr..

[49] Guleria S., Tiku A.K., Rana S. (2011). Antioxidant and free radical scavenging activity of acetone extract fractions of *Terminalia belleria* Roxb fruit. Indian J. Biochem. Biophys..

[50] Heinz-Peter S. (1989). The chemical basis of radiation biology: By C. von Sonntag; published by Taylor and Francis, London. pp. 515. J. Photochem. Photobiol B Biol..

[51] Tapiero H., Townsend D.M., Tew K.D. (2003). Trace elements in human physiology and pathology. Biomed. Pharmacother..

[52] Linder M.C. (1991). Introduction and Overview of Copper as an Element Essential for Life. Biochemistry of Copper. Biochemistry of the Elements.

[53] Zhou J., Kang H.M., Lee Y.H., Jeong C.B., Park J.C., Lee J.S. (2019). Adverse effects of a synthetic pyrethroid insecticide cypermethrin on life parameters and antioxidant responses in the marine copepods *Paracyclopina nana* and *Tigriopus japonicus*. Chemosphere.

[54] Arif A., Salam S., Mahmood R. (2020). Bioallethrin-induced generation of reactive species and oxidative damage in isolated human erythrocytes. Toxicol. In Vitro.

[55] Uttara B., Singh A.V., Zamboni P., Mahajan R.T. (2009). Oxidative stress and neurodegenerative diseases: A review of upstream and downstream antioxidant therapeutic options. Curr. Neuropharmacol..

[56] Punia S., Kaur J., Kumar R., Kumar K. (2014). *Catharanthus roseus*: A medicinal plant with potent anti-tumor properties. Int. J. Res. Ayurveda Pharm..

[57] Halsted C.H., Villanueva J.A., Devlin A.M., Niemela O., Parkkila S., Garrow T.A., Wallock L.M., Shigenaga M.K., Melnyk S., James S.J. (2002). Folate deficiency disturbs hepatic methionine metabolism and promotes liver injury in the ethanol-fed micropig. Proc. Natl. Acad. Sci. USA.

[58] Pravenec M., Kozich V., Krijt J., Sokolová J., Zídek V., Landa V., Simáková M., Mlejnek P., Silhavy J., Oliyarnyk O. (2013). Folate deficiency is associated with oxidative stress, increased blood pressure, and insulin resistance in spontaneously hypertensive rats. Am. J. Hypertens..

[59] Trotta R.J., Sullivan S.G., Stern A. (1983). Lipid peroxidation and haemoglobin degradation in red blood cells exposed to t-butyl hydroperoxide. The relative roles of haem- and glutathione-dependent decomposition of t-butyl hydroperoxide and membrane lipid hydroperoxides in lipid peroxidation and haemolysis. Biochem. J..

[60] Tesoriere L., D’Arpa D., Butera D., Pintaudi A.M., Allegra M., Livrea M.A. (2002). Exposure to malondialdehyde induces an early redox unbalance preceding membrane toxicity in human erythrocytes. Free Radical Res..

[61] Puppo (1988). A.; Hallwell. B. Formation of hydroxyl radicals from hydrogen peroxide in the presence of iron. Biochem. J..

[62] Tsou T.C., Yang J.L. (1996). Formation of reactive oxygen species and DNA strand breakage during interaction of chromium (III) and hydrogen peroxide in vitro: Evidence for a chromium (III)-mediated Fenton-like reaction. Chem. Biol. Interact..

[63] Chater S., Douki T., Garrel C., Favier A., Sakly M., Abdelmelek H. (2008). Cadmium-induced oxidative stress and DNA damage in kidney of pregnant female rats. Comptes Rendus Biol..

[64] Snezhkina A.V., Kudryavtseva A.V., Kardymon O.L., Savvateeva M.V., Melnikova N.V., Krasnov G.S., Dmitriev A.A. (2019). ROS Generation and Antioxidant Defense Systems in Normal and Malignant Cells. Oxid. Med. Cell. Longev..

